# Two-Week Recovery Strategies to Enhance Performance Readiness in Martial Arts Athletes: A Pilot Study

**DOI:** 10.3390/sports14020046

**Published:** 2026-02-02

**Authors:** Behnam Boobani, Juris Grants, Sergejs Saulite, Germans Jakubovskis, Anna Zusa, Edgars Bernans, Žermēna Vazne, Katrina Volgemute, Marta Stromberga, Artur Litwiniuk

**Affiliations:** 1 RSU Latvian Academy of Sport Education, Riga Stradins University, LV-1006 Riga, Latvia; juris.grants@rsu.lv (J.G.); sergejs.saulite@rsu.lv (S.S.); 2 Sports Healthcare Research Center, Riga Stradins University, LV-1006 Riga, Latvia; germans.jakubovskis@rsu.lv (G.J.); anna.zusa@rsu.lv (A.Z.); edgars.bernans@rsu.lv (E.B.); 3 Department of Health Psychology and Pedagogy, Riga Stradins University, LV-1007 Riga, Latvia; zermena.vazne@rsu.lv (Ž.V.); katrina.volgemute@rsu.lv (K.V.); 4 Department of Human Physiology and Biochemistry, Riga Stradins University, LV-1007 Riga, Latvia; martastromberga@rsu.edu.lv; 5 Faculty of Physical Education and Health, Jozef Pilsudski University of Physical Education in Warsaw, 21-500 Biala Podlaska, Poland; a.litwiniuk@wp.pl

**Keywords:** taekwondo athletes, recovery methods, performance

## Abstract

This study aimed to examine preliminary responses of two-week post-exercise recovery strategies on performance readiness in well-trained Taekwondo athletes. Fifteen athletes were randomly assigned to cryotherapy (partial-body cryotherapy followed by cold-water immersion; *n* = 5 per group), foam rolling (FR; *n* = 5 per group), and control (CON; *n* = 5 per group). The intervention lasted two weeks and consisted of post-exercise recovery strategies only. Performance and recovery outcomes were assessed using the Latvian Recovery–Stress Questionnaire (RESTQ), the determination test of the Vienna test (DT), the countermovement jump (CMJ), and isokinetic knee flexion and extension. Data were analyzed using mixed-design ANOVA. Significant time effects were observed for DT (F(1,12) = 5.91, *p* = 0.03, η^2^p = 0.33), CMJ (F(1,12) = 12.44, *p* = 0.004, η^2^p = 0.50), and knee extension (F(1,12) = 5.20, *p* = 0.04, η^2^p = 0.30). No changes were detected under the present conditions for RESTQ stress and recovery scores and knee flexion (*p* > 0.05). Overall, the findings indicate time-dependent changes in several performance outcomes, while differences between recovery conditions should be interpreted as exploratory, as no clear intervention-specific effects were demonstrated under the study conditions.

## 1. Introduction

Athletes in many sports follow intensive training programs to achieve peak performance during major competitions. Taekwondo is a high-intensity combat sport characterized by rapid and forceful actions, including powerful kicks, fast punches, and dynamic defensive movements [1,2]. Front and roundhouse kicks are often performed with jumping and rotational elements, requiring high explosive capacity, and can attain speeds of 5.2–18.3 m/s [3,4]. Competitive bouts involve repeated short-duration offensive and defensive exchanges, most lasting less than 2 s [5,6]. At national and international tournaments, athletes often compete in multiple matches per day, leaving little time for recovery. These combined physical and psychological demands place substantial stress on athletes during training and competition. Under such congested competition schedules, neuromuscular performance (e.g., strength and power output), cognitive functions related to reaction speed and stress tolerance, and perceived stress–recovery balance are particularly susceptible to fatigue-related impairment. To achieve and sustain high performance, athletes engage in demanding training sessions [7] that rely on the combined contribution of strength, speed, and multidirectional movement abilities, which are essential for repeated kicking actions in Taekwondo [8,9]. Such training loads can induce biological and mechanical stress, resulting in temporary impairments in muscle structure, functional capacity, and overall performance [10,11]. Consequently, recovery strategies are frequently applied after intensive training and during competitions with short recovery intervals, although their effects on fatigue and performance are highly context-dependent and influenced by sport demands, training load, outcome measures, and timing [11].

Post-exercise recovery focuses on processes that restore function between training sessions or competitions [12] and lessen the adverse impacts of training on biochemical, physiological, and physical performance in combat sport athletes [5,13]. Implementing effective recovery strategies is essential for sustaining high-level performance and minimizing negative outcomes [14]. In recent years, several recovery modalities, including massage, cold exposure, and infrared therapies, have demonstrated greater effectiveness than passive recovery in restoring physiological and functional capacities [15]. Beyond traditional massage, self-myofascial techniques, such as foam rolling, have become widely adopted in sport and fitness settings and are now among the most commonly used recovery tools among athletes [16]. However, evidence regarding the effectiveness of recovery modalities such as cryotherapy and massage-based interventions remains mixed and inconsistent [15,17,18,19,20,21,22,23,24,25], and current knowledge does not allow firm conclusions, particularly for well-trained athletes. This heterogeneity in findings underscores the need for exploratory pilot research.

Cryotherapy-based interventions help to reduce delayed-onset muscle soreness (DOMS) following high-intensity training in combat sports. Exposure to temperatures (−110 to −130 °C) is well tolerated by professional athletes and may attenuate exercise-induced inflammation while supporting performance during intensive training and competition periods [26]. However, evidence regarding the effects of cryotherapy on performance remains inconclusive [17,18]. Studies have shown that countermovement jump impairments following cryotherapy in martial arts athletes [20,21] and exposure to cold can temporarily decrease force production due to a drop in muscle temperature [22].

Taekwondo is governed internationally by two main organizations; while World Taekwondo has been extensively examined in the scientific literature, research focusing on the International Taekwondo Federation remains limited [27,28]. ITF Taekwondo differs from World Taekwondo in its scoring system and in permitting hand strikes to the head, which may alter bout dynamics, tactical decision-making, and the relative contribution of upper- and lower-limb actions during competition [27]. The sport’s metabolic profile is characterized by repeated high-intensity, short-duration kicking actions, requiring training that emphasizes maximal force, high velocity, and frequent execution. Consequently, athletes must sustain repeated powerful attacks over time while minimizing fatigue-related declines in performance [29,30,31,32].

Knowledge regarding recovery strategies specifically suited to ITF Taekwondo athletes remains limited. This study is intended as a pilot investigation to explore the feasibility, variability, and preliminary performance responses to different recovery strategies in combat sports contexts [33,34], particularly during training and before competitions, when athletes are required to complete multiple bouts within short intervals.

The effects of post-exercise recovery strategies on performance readiness in ITF Taekwondo athletes remain unexamined. Therefore, the present study was designed as a pilot investigation to explore the feasibility, variability, and preliminary performance responses associated with two weeks of different recovery interventions, including combined cryotherapy (partial-body cryotherapy followed by cold-water immersion; PBC/CWI), foam rolling, and passive recovery. Performance readiness was assessed using multiple outcome measures, including stress and recovery score of the recovery–stress questionnaire (RESTQ), reactive stress tolerance of the determination test (DT), countermovement jump (CMJ), and isokinetic knee strength, in well-trained ITF athletes. It was hypothesized that performance readiness indicators would exhibit variable and exploratory response patterns across recovery strategies.

## 2. Materials and Methods

### 2.1. Participants

Fifteen well-trained ITF Taekwondo athletes (6 females, 9 males) from Riga, Latvia (Table 1), volunteered to take part. To be eligible, participants should have participated in national or international Taekwondo competitions, have five years of training, and attend four training sessions per week. Athletes were excluded if they reported pharmacological medication use, cold intolerance, cardiovascular or neurological conditions, or current musculoskeletal injuries. Participants were aged 14–21 years (M = 16.26, SD = 2.86), with a mean height of 170.8 cm (SD = 5.66) and body mass of 63.96 kg (SD = 7.40). Anthropometric measurements were obtained using standardized equipment (Seca 213 and 813, Seca GmbH, Hamburg, Germany). All athletes were classified as level 3 (highly trained) [35]. The availability of athletes determined the sample size. The relatively small sample size and wide age range are consistent with the pilot nature of the study, which was primarily exploratory. Consequently, potential sex- and age-related differences could not be formally examined due to limited statistical power. Baseline characteristics were therefore summarized descriptively, and no inferential comparisons between groups at baseline were conducted. Accordingly, no a priori or post hoc power analysis was performed, as the study was designed as an exploratory pilot focusing on feasibility and variability. The study followed the Declaration of Helsinki, and ethical approval was obtained from the Ethical Committee of the Latvian Academy of Sport Education (protocol no. 9/3, 31 May 2024). All athletes signed informed consent.

### 2.2. Instruments and Procedures

The study protocol consisted of baseline (pre-test) and post-intervention assessments conducted two weeks apart. The two-week intervention period was chosen to emphasize feasibility and sensitivity to short-term recovery-related changes in performance readiness, rather than longer-term adaptations, consistent with the exploratory pilot design of the study. Participants were familiarized with all testing and recovery procedures one week prior to baseline measurements. At both time points, performance readiness was assessed using a battery that included the recovery–stress questionnaire (RESTQ) in Latvian [36,37], the Determination test (DT), the countermovement jump (CMJ), and isokinetic knee strength (flexion and extension). All assessments were conducted between 15:00 and 17:00 at the sports healthcare research center of the Latvian Academy of Sport Education, Riga Stradins University. Participants were instructed to avoid alcohol, caffeine, and strenuous physical activity for 24 h before testing and verbally confirmed compliance.

#### 2.2.1. RESTQ

The RESTQ questionnaire is based on a 7-point Likert scale (0 = never to 6 = always) to measure stress and recovery over the three days and nights [36,38]. Although the instrument comprises multiple subscales, the present pilot study focused on aggregated total stress and total recovery scores due to sample size limitations and the exploratory aim; therefore, subscale-level analyses were not conducted. Test–retest reliability of the Latvian RESTQ was acceptable (ICC = 0.66–0.78 over 10 days) [38].

#### 2.2.2. DT

The determination test is a computerized assessment commonly used to evaluate cognitive performance and reaction-stress tolerance in sport settings [39]. During the DT, participants respond as quickly and accurately as possible to visual and auditory stimuli using both hands and feet, requiring stimulus discrimination, response selection, and motor execution. These demands are relevant to Taekwondo competition, where athletes must rapidly perceive external cues, make decisions under time pressure, and execute appropriate actions while managing high levels of cognitive and emotional stress [39]. Accordingly, DT outcomes were selected as indicators of performance readiness, reflecting decision-making under pressure and stress tolerance relevant to Taekwondo performance. Test–retest reliability of the DT has been reported as good (r = 0.90 over 7–14 days) [40]. The test duration was approximately 3–5 min.

#### 2.2.3. CMJ

An Optojump (Microgate^®^, Bolzano, Italy) was used to measure the CMJ. Athletes performed jumps with hands on hips and a standardized countermovement depth of approximately 90° knee flexion under verbal instruction [1]. Three trials were completed with 15 s rest intervals, and the highest jump was used for the analysis. CMJ assessed using the OptoJump has demonstrated excellent test–retest reliability (ICC = 0.98) [41].

#### 2.2.4. Isokinetic Knee Strength (Flexion and Extension)

Maximal torque during knee flexion and extension was assessed using an isokinetic dynamometer (Con-Trex MJ, Physiomed, Schnaittach, Germany), calibrated according to the manufacturer’s guidelines. Following the warm-up, athletes were seated and secured with chest, hip, and thigh straps. The dominant leg was identified based on the self-reported kicking leg [42]. Maximal torque was measured at an angular velocity of 60°·s^−1^, three times under standardized verbal instruction and encouragement as previously applied to Taekwondo athletes [42]. Higher angular velocities, which may more closely reflect the ballistic nature of kicking actions, were not assessed to limit testing burden within the exploratory study design. Isokinetic knee strength, assessed with the Con-Trex dynamometer, has demonstrated moderate-to-excellent test–retest reliability (ICC > 0.86) [43].

All assessments were conducted by members of the research team, who were not blinded to group allocation due to the practical constraints of the pilot study design.

### 2.3. Recovery Interventions

Athletes with national and international competitive experience were represented across all groups; competitive level was not used as a stratification factor. Athletes were allocated to one of three groups: combined cryotherapy (partial-body cryotherapy followed by cold-water immersion; PBC/CWI, *n* = 5 per group), foam rolling (FR, *n* = 5 per group), or passive recovery control (CON, *n* = 5 per group). The intervention lasted two weeks. During week 1, athletes in the cryotherapy group underwent PBC following four Taekwondo training sessions using a cryotherapy chamber (CTN Cryo Cabin, Helsinki, Finland). Participants prepared by drying their skin, removing metal items, and wearing protective gloves and footwear before exposure to vaporized liquid nitrogen at −110 °C for 3 min while standing upright with the head positioned outside the chamber. In week 2, the same athletes completed CWI after each training session by sitting in a tank bath at 10 °C for 20 min, as previously described in Taekwondo research [44]. Water temperature was monitored and adjusted, and participants were instructed to periodically move their legs to ensure uniform cooling [45]. The combined PBC/CWI protocol limits the ability to isolate modality-specific effects; however, this approach was chosen to maximize feasibility and ecological validity within an exploratory pilot design, reflecting applied recovery practices rather than mechanistic investigation.

The foam-rolling protocol was performed using a compact foam roller (Sveltus, Le Chambon-Feugerolles, France) and targeted the quadriceps, hamstrings, adductors, and gastrocnemius [46]. Athletes completed three 30 s sets per muscle group, with 30 s rest intervals. Rolling was performed through controlled proximal-to-distal movements while maintaining standardized body positions appropriate for each muscle group. The selected muscle groups and rolling volume were chosen based on their relevance to Taekwondo lower-limb actions and consistency with previously published protocols [46]. The control group performed passive seated recovery for 20 min, consistent with control conditions used in previous Taekwondo studies [44]. Adherence to the recovery protocols was monitored, and all participants completed the prescribed interventions.

The Taekwondo training program remained unchanged throughout the study and was conducted four times per week at the Latvian Academy of Sport Education over two weeks (eight sessions, 90 min each). Sessions included a standardized warm-up, followed by kicking, technical, and sparring exercises, and a plyometric training consisting of jumps from a 60 cm box [47]. All training was performed at approximately 85% of predicted maximal heart rate, monitored via pulse oximetry and estimated using established age-based equations [48]. Training load was standardized pragmatically rather than precisely controlled, acknowledging the limitations of age-predicted heart rate estimation.

### 2.4. Statistical Analysis

Data analysis was performed using SPSS (version 26.0; IBM Corp; Armonk, NY, USA) and JASP (version 0.18.3). Results are presented as means ± standard deviations. Data distribution and homogeneity of variance were assessed using the Shapiro–Wilk and Levene’s tests; assumptions were met for all dependent variables. A mixed-design ANOVA with Time (pre vs. post) and Group (cryotherapy, foam rolling, control) followed by Bonferroni-adjusted post hoc comparisons. Given the small sample size, this analysis was conducted within an exploratory pilot framework to examine overall time-dependent patterns rather than to establish definitive intervention effects. Effect sizes are reported as partial eta squared (η^2^p), and interpreted using conventional thresholds (small ≥ 0.01, medium ≥ 0.06, large ≥ 0.14) [18]. Statistical significance was set at *p* < 0.05.

## 3. Results

**Figure 1:**
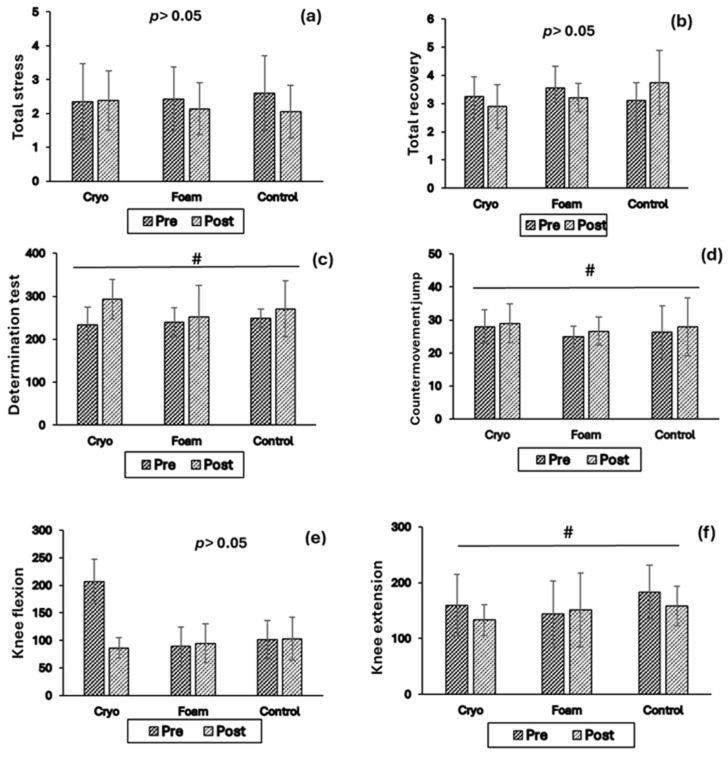
Comparison of the analyzed parameters in pre- and post: #: significant main effect of time (*p* < 0.05), for panels (**a**,**b**,**e**), *p* > 0.05: no significant time × group interaction, (**a**): total stress, (**b**): total recovery, (**c**): determination tests, (**d**): countermovement jump, (**e**): knee flexion, (**f**): knee extension.

Table 2 summarizes descriptive statistics and 95% confidence intervals for all groups; the wide intervals, which frequently overlapped zero, reflect high variability and imprecision in the pilot sample and warrant cautious interpretation of the point estimates. For RESTQ total stress and total recovery scores (Figure 1a,b), mixed-design ANOVA did not indicate differential changes between recovery groups over time. Specifically, no Time × Group interaction was observed for total stress (F(2,12) = 1.03, *p* = 0.38, η^2^p = 0.14) or total recovery (F(2,12) = 2.62, *p* = 0.11, η^2^p = 0.09). Main effects of Time (stress: F(1,12) = 2.49, *p* = 0.14; recovery: F(1,12) = 0.001, *p* = 0.97) and Group (stress: F(2,12) = 0.01, *p* = 0.99; recovery: F(2,12) = 0.39, *p* = 0.68) were also not evident. Overall, RESTQ stress and recovery scores remained relatively stable over the two weeks. Given the small sample size, these null findings should be interpreted cautiously and are reported to describe observed patterns rather than to infer equivalence between recovery conditions.

Analysis of DT (Figure 1c) showed a significant main effect of Time, F(1,12) = 5.91, *p* = 0.03, η^2^p = 0.33. However, individual responses varied, and the observed Time effect was evident across all groups rather than being specific to any recovery condition. No significant Time × Group interaction was observed, F(2,12) = 1.29, *p* = 0.30, η^2^p = 0.17, and no significant differences were found between groups, F(2,12) = 0.22, *p* = 0.80, η^2^p = 0.03.

For CMJ (Figure 1d), there was a significant effect of Time, F(1,12) = 12.44, *p* = 0.004, η^2^p = 0.50, indicating increased jump height from pre- to post-test. Descriptively, CMJ increased in all groups, indicating that the observed Time effect was not specific to any recovery intervention. No significant Time × Group interaction or Group main effect was observed (*p* > 0.05).

For knee flexion torque (Figure 1e), no significant effects of Time, Group, or their interaction were observed (*p* > 0.05). In contrast, knee extension torque (Figure 1f) showed a significant main effect of Time, F(1,12) = 5.20, *p* = 0.04, η^2^p = 0.30, indicating an overall decrease from pre- to post-test. Descriptively, this decrease was observed across all groups, indicating that the Time effect was not specific to any recovery intervention. No significant Time × Group interaction or Group main effect was observed (*p* > 0.05).

In summary, mixed-design ANOVA identified significant main effects of Time for DT, CMJ, and knee extension torque, while no significant Time × Group interactions or Group main effects were observed for any outcome. These findings indicate that observed changes occurred over time but did not differ between recovery strategies. All reported outcomes were analyzed exploratively, with no outcome designated as primary, reflecting the pilot design and feasibility-focused aims of the study.

## 4. Discussion

This study explored the effects of two weeks of post-exercise recovery strategies, cryotherapy (PBC/CWI), foam rolling, and passive recovery, on performance readiness in well-trained ITF Taekwondo athletes. The main findings indicated significant time-dependent changes in selected performance outcomes, including improvements in DT and CMJ performance and a decrease in knee extension torque, whereas no significant changes were observed in RESTQ stress and recovery scores or in knee flexion. Accordingly, the present findings should be interpreted as exploratory, reflecting short-term temporal responses rather than evidence of differential effectiveness between recovery strategies and passive recovery. The RESTQ results align with the scissors model [36], which conceptualizes stress and recovery as interdependent processes. Previous RESTQ-based studies have similarly reported stable stress–recovery profiles across training and competition phases [49]. In contrast, research on martial arts athletes has shown improved perceived recovery following massage or cold-based interventions, likely due to methodological differences, including repeated post-exercise assessments and the use of alternative recovery measures, such as the total quality recovery scale (TQR) [50]. Recovery responses are highly individual and influenced by personal perception [51,52]. It is therefore possible that non-training stressors, such as academic or social demands, may have contributed to variability in perceived recovery in the present sample; however, these factors were not directly measured and therefore remain speculative. The overall improvement in DT performance may reflect nonspecific time-related factors rather than intervention-specific effects. Although the arousal hypothesis suggests that cold exposure can transiently enhance alertness and cognitive function [53], DT improvements were observed across all groups, and no significant Time × Group interaction was detected. Accordingly, the observed time effect cannot be attributed specifically to cryotherapy or foam rolling. Similar improvements in cognitive performance during repeated testing have been reported previously and are commonly attributed to learning or familiarization effects rather than recovery-related adaptations [54]. Foam rolling has also been suggested to reduce perceived stress or fatigue, which could indirectly support cognitive readiness [55]; however, given the absence of group differences in the present study, such interpretations remain speculative. The observed DT changes should therefore be interpreted as general temporal responses within an exploratory pilot framework. CMJ performance improved over time across all groups. This finding is consistent with reports of time-dependent improvements in CMJ during repeated cryotherapy exposure [15,19], but contrasts with studies showing CMJ impairments following cryotherapy in martial arts athletes [20,21]. Foam rolling has been associated with enhanced jump performance; however, under the present conditions and over the two weeks, it was not associated with additional improvements beyond those observed across groups. Given the pilot design and limited statistical power, these observations should be interpreted cautiously and considered exploratory rather than indicative of modality-specific effects. Variations in study design and intervention timing may explain these inconsistencies.

The knee flexion did not change significantly, whereas knee extension decreased over time. This decrease may reflect accumulated neuromuscular fatigue associated with repeated high-intensity training; however, neuromuscular fatigue was not directly assessed in the present study. Cold exposure may transiently impair force production by reducing muscle temperature, impairing neural conduction, and attenuating anabolic signaling involved in neuromuscular adaptation [22]. The absence of strength gains following cryotherapy may also relate to the applied temperature and exposure duration. Evidence regarding massage-based recovery remains inconsistent, with studies reporting both reductions and improvements in knee extension torque following foam rolling [23,24,25]. Individual differences may influence such discrepancies in applied pressure, discomfort tolerance, and training status, as recovery-related performance effects appear more pronounced in untrained populations than in well-trained athletes [56]. Direct comparison with the present pilot findings is limited because applied pressure was not objectively quantified in our protocol; rolling volume and timing vary widely across studies; populations often differ (untrained vs. well-trained athletes); and outcome measures are not comparable (alternative strength tests vs. isokinetic knee extension torque) [56].

Although the effectiveness of both interventions appears to depend on methodological factors such as exercise type, treatment duration, and assessment timing, which may explain inconsistent findings in Taekwondo research [16,23,57], several limitations should be considered. The small sample size and the study’s pilot nature as primary limitationslikely reduced the power to identify group-level differences [58]. The use of a pre–post design without intermediate assessments limited insight into fluctuations in short-term recovery. RESTQ, as a self-reported measure, might have led to response bias. Additionally, the combined cryotherapy protocol prevented the separation of the specific effects of PBC and CWI. The absence of objective physiological biomarkers of recovery, and uncontrolled factors such as sleep, nutrition, and external stressors, may have influenced perceptions of recovery.

## 5. Conclusions

This study evaluated the effects of two weeks of post-exercise recovery strategies on multiple performance-related outcomes in well-trained ITF Taekwondo athletes. While several variables, including DT and CMJ, improved over time, knee extension decreased, and no significant changes were observed in stress–recovery (RESTQ) or knee flexion. No intervention-specific effects were identified, and the observed changes should be interpreted as short-term, time-dependent responses within the context of this pilot study. Accordingly, the present findings reflect feasibility and preliminary response patterns rather than evidence of recovery strategy efficacy. These results are specific to well-trained ITF Taekwondo athletes and should be interpreted within that context rather than generalized to other combat sports, training levels, or competitive contexts. Future research should build on these pilot findings to inform study design, including sample size estimation, refinement of recovery protocols, and the inclusion of objective physiological markers to characterize recovery-related mechanisms in this population better.

## Figures and Tables

**Table 1:** Participants’ group characteristics.

	Group (*n* = 15)		
	Cryotherapy *n* = 5	Foam Rolling *n* = 5	Control *n* = 5
	m ± SD		
Age (years)	16.6 ± 3.57	15.8 ± 2.95	16.4 ± 2.6
Height (cm)	169.7 ± 5.19	171.6 ± 6.72	171.1 ± 6.15
Weight (kg)	63.04 ± 4.72	66.02 ± 10.66	62.82 ± 6.91
Body mass index (kg/m^2^)	21.88 ± 1.31	22.31 ± 2.44	21.43 ± 1.73

cm: centimeter; kg: kilogram.

**Table 2:** Descriptive statistics and 95% CIs of groups.

Variable	Test Phase	Cryotherapy *n* = 5 m ± SD 95% CI Lower, Upper	Foam Rolling *n* = 5 m ± SD 95% CI Lower, Upper	Control *n* = 5 m ± SD 95% CI Lower, Upper
RESTQ total stress	Pre	2.34 ± 1.13 (0.93 ± 3.75)	2.43 ± 0.95 (1.25 ± 3.61)	2.63 ± 1.11 (1.25 ± 4.01)
	Post	2.38 ± 0.88 (1.28 ± 3.48)	2.14 ± 0.77 (1.17 ± 3.10)	2.05 ± 0.78 (1.07 ± 3.02)
RESTQ total recovery	Pre	3.24 ± 0.70 (2.37 ± 4.11)	3.55 ± 0.78 (2.58 ± 4.51)	3.11 ± 0.63 (2.32 ± 3.89)
	Post	2.91 ± 0.77 (1.95 ± 3.87)	3.21 ± 0.50 (2.58 ± 3.84)	3.75 ± 1.13 (2.34 ± 5.16)
DT	Pre	233.80 ± 41.61 (182.13 ± 285.47)	239.80 ± 33.53 (198.15 ± 281.44)	249 ± 21.77 (221.96 ± 276.03)
	Post	293.20 ± 45.61 (236.56 ± 349.83)	251.80 ± 73.81 (160.15 ± 343.45)	270.40 ± 65.26 (189.36 ± 351.43
CMJ (cm)	Pre	27.98 ± 5.11 (21.62 ± 34.33)	24.86 ± 3.17 (20.91 ± 28.80)	26.22 ± 7.97 (16.31 ± 36.12)
	Post	28.96 ± 5.89 (21.63 ± 36.28)	26.60 ± 4.35 (21.19 ± 32)	27.84 ± 8.78 (16.92 ± 38.75)
Isokinetic knee flex (Nm)	Pre	107.24 ± 40.44 (57.02 ± 157.45)	89.08 ± 35.39 (45.13 ± 133.03)	101.64 ± 34.32 (59.01 ± 144.26)
	Post	86.28 ± 18.75 (62.99 ± 109.56)	94.64 ± 35.30 (50.81 ± 138.47)	103.30 ± 39.08 (54.76 ± 151.83)
Isokinetic knee ext (Nm)	Pre	159.84 ± 54.60 (92.03 ± 227.64)	143.94 ± 59.10 (70.55 ± 217.32)	183.56 ± 47.75 (124.26 ± 242.85)
	Post	133.08 ± 27.57 (98.84 ± 167.31)	151.38 ± 65.91 (69.53 ± 233.22)	158.48 ± 35.77 (114.06 ± 202.89)

m: mean, SD: standard deviation; CI: confidence interval; RESTQ: recovery–stress questionnaire, DT: determination test; CMJ: countermovement jump; cm: centimeter; flex: flexion.; ext: extension; Nm: newton meter.

## Data Availability

The original contributions presented in the study are included in the article, further inquiries can be directed to the corresponding author/s.
